# Prevalence and Risk Factors of Anxiety in a Clinical Dutch Sample of Children with an Autism Spectrum Disorder

**DOI:** 10.3389/fpsyt.2018.00050

**Published:** 2018-03-02

**Authors:** Lieke A. M. W. Wijnhoven, Daan H. M. Creemers, Ad A. Vermulst, Isabela Granic

**Affiliations:** ^1^Mental Health Care Institute GGZ Oost-Brabant, Boekel, Netherlands; ^2^Behavioural Science Institute, Radboud University, Nijmegen, Netherlands

**Keywords:** prevalence, risk factors, anxiety symptoms, children, autism spectrum disorders

## Abstract

Anxiety is highly prevalent in children with an autism spectrum disorder (ASD). However, there is inconsistency in studies investigating the prevalence and risk factors of anxiety in children with ASD. Therefore, the first aim of this study was to give an overview of the prevalence of anxiety symptoms in a clinical Dutch sample of children with ASD. The second aim was to investigate age, gender, ASD subtype, and IQ as potential risk factors for anxiety in this sample. In total, 172 children with ASD (age, 8–15 years) and their parents participated in this study. Specialized services in which children with ASD were recruited were two mental health institutes and one secondary special education school. The findings showed that more than 60% of the participating children with ASD had at least subclinical anxiety symptoms according to children. More than 80% of the children with ASD had at least subclinical anxiety symptoms according to parents. It was found that younger children and girls with ASD had more anxiety symptoms than older children and boys with ASD. Moreover, it was found that children with a higher performance (non-verbal) IQ and lower verbal IQ had more specific phobia symptoms. The findings suggest that in a clinical context, children with ASD have a high risk to have co-occurring anxiety symptoms, especially girls and younger children with ASD. Therefore, early prevention and treatment of anxiety in children with ASD who are most at risk is important.

## Introduction

Recent studies have demonstrated that comorbid psychiatric problems are common among children with an autism spectrum disorder [ASD; e.g., Ref. (1)]. Anxiety is one of the most prevalent comorbid psychiatric problems in children with ASD, with prevalence rates varying between 11 and 84% (2). More specifically, it has been shown that 56% of the children with an ASD suffer from at least subclinical anxiety (3), and approximately 40% of the children with an ASD meet the criteria of at least one anxiety disorder (4). Some of the most frequently reported anxiety disorders and symptoms seen in children with an ASD are specific phobias, generalized anxiety disorder, separation anxiety disorder, obsessive–compulsive disorder, and social phobia (2).

The distinct anxiety disorders show a wide variation in prevalence rates [11–84% (2)] in the population of children with ASD. The big differences in prevalence rates are mainly due to sample and methodological heterogeneity, differences in the assessment of anxiety in children with ASD, and differences in the operationalization of anxiety (5). In some studies, only parent report of anxiety was used [e.g., Ref. (6)], whereas in other studies, a combination of parent and child [e.g., Ref. (7)] or parent and teacher (8) report of anxiety was used. These respondents differ in their assessment of anxiety in children with ASD. For example, there is some evidence that teachers give higher ratings of anxiety than parents (9). Moreover, some children with ASD may have difficulties with emotional insight or accurately detecting and interpreting one’s own emotions (2), which could lead to higher symptom ratings of parents compared to children. However, other children with ASD might be able to report on their own stress and social attributions (10). For example, ASD children with age appropriate intellectual and verbal abilities have relatively intact emotion recognition abilities (11). This implicates that some children with ASD may at least be comparable to parents in their report of anxiety symptom levels. Furthermore, anxiety could be conceptualized as anxiety symptoms measured by a questionnaire (7), but also as anxiety disorders measured by a structured interview (12). The use of interviews is associated with higher prevalence rates of total anxiety, while for social anxiety disorder, the use of questionnaires is associated with higher prevalence rates (4). These conceptual and methodological differences and the heterogeneity of the ASD population may lead to variation in the prevalence rates across studies.

Because anxiety causes significant impairment in children with ASD, it is important that efforts are made to improve the identification and assessment of these symptoms. The impairments caused by anxiety consist of poor daily living skills and problems in relationships with peers, teachers, and family (13–15). Moreover, anxiety underlies comorbid symptoms of children with an ASD, for example, oppositional and aggressive behavior (16) and depressive symptoms (17). The impairments and comorbid symptoms caused by anxiety also underline the importance of effective treatment of anxiety and prevention of further escalation.

To be able to prevent further escalation of anxiety, it is important that risk factors that predict the development of anxiety are identified. In this way, children with ASD can be profiled on vulnerability for the development of anxiety. Moreover, prevention and treatment can specifically be tailored to the ASD children with elevated risk to develop anxiety. In the literature, several potential but inconsistent risk factors for the development of anxiety in children with ASD are identified. Again, the inconsistency in risk factors is often due to conceptual and methodological differences between studies and the heterogeneity of the ASD population (5).

Age is one of the potential but inconsistent risk factors. Most studies reported that anxiety in children with ASD increases from preschool to adolescence [e.g., Ref. (18, 19)]. Potentially, the delayed cognitive and motor developments in children with ASD do not permit the recognition and expression of anxiety symptoms until adolescence (18). However, there is also evidence that only clinical anxiety is higher in adolescents with ASD compared to school children with ASD and that subclinical anxiety is higher in school children with ASD compared to adolescents with ASD (17).

There are also mixed data regarding the differential prevalence rates of anxiety for girls and boys with ASD. Some studies showed that girls with ASD have more anxiety symptoms (9), and other studies showed that anxiety is more prevalent in boys with ASD (20). There are more studies showing that anxiety symptoms are equally prevalent in boys and girls with ASD (17, 21, 22). This could be explained by the hypothesis that the shared neurobiological dysfunctions in boys and girls with ASD have an overriding effect on psychopathology, leading to comparable anxiety levels in boys and girls with ASD (23).

Moreover, several studies showed that the prevalence of anxiety varies among the different subtypes of ASD. The studies that specifically focused on comparing anxiety levels across ASD subtypes suggest that ASD severity is associated with the level of anxiety and that children with less severe ASD symptoms might be most comorbid with anxiety (24). Specifically, children with Asperger disorder experience more anxiety than children with PDD-NOS, and children with PDD-NOS experience more anxiety than children with autistic disorder (2, 9, 25). This is in line with research that showed that higher IQ or better cognitive functioning predicted more severe anxiety in children with ASD [e.g., Ref. (1, 17, 20)], because children with Asperger disorder have a higher IQ than children with PDD-NOS and autistic disorder [e.g., Ref. (26)]. Higher IQ and developmental level may allow children with Asperger disorder to engage more in higher order cognitions (e.g., worry, foresight, and imagery), which are the hallmark of several anxiety disorders (5). Moreover, it is possible that children with a higher IQ are more exposed to situations (e.g., interactions with peers) that provoke social anxiety (1). Finally, children with Asperger disorder have better verbal capacities than children with PDD-NOS and autistic disorder, which supports the communication of anxiety to parents and teachers (1).

Conversely, there is some evidence that anxiety in general is more common in children with PDD-NOS, followed by children with autistic disorder and Asperger disorder (4). Moreover, Dickerson Mayes et al. (19) found that anxiety increased with autism severity. In line with this, some studies reported that a lower mean IQ of children with ASD is associated with higher prevalence rates of anxiety [e.g., Ref. (4)]. Because children with PDD-NOS and autistic disorder have more severe ASD symptoms than children with Asperger disorder, they may experience more ASD-related anxiety, for example, anxiety for unexpected social situations (4).

Furthermore, the verbal IQ (VIQ) and performance (non-verbal) IQ (PIQ) should be considered as more specific risk factors for anxiety in children with ASD. PIQ is often relatively high and VIQ relatively low in children with ASD (27). This is consistent with the core ASD symptom of communication impairments, which may be reflected in weak verbal capacities in children with ASD. Unlike in the normal population, impairments in communication may have an inverse relationship with anxiety in children with ASD. For example, deficits in both receptive and expressive communication skills are associated with less anxiety symptoms in children with ASD (28). In line with this finding, there is some evidence that ASD children with lower non-verbal and higher verbal skills show more anxiety and mood problems, which would mean that a low PIQ and high VIQ are related to more anxiety in children with ASD (15). However, other research showed that children with PDD-NOS had higher anxiety levels as communication deficits increased (29).

It can be concluded that there is inconsistency in results of studies investigating prevalence and risk factors of anxiety in children with ASD. Therefore, the first aim of this study was to give an overview of the prevalence of anxiety symptoms in a clinical Dutch sample of children with ASD. The second aim was to investigate the risk factors for anxiety in this sample of children with ASD. More specifically, age, gender, ASD subtype, and IQ were examined as potential risk factors for anxiety in children with ASD. We expected that older children with ASD experience more anxiety than younger children with ASD and that there was no difference in anxiety between boys and girls with ASD. Also, we expected that children with Asperger disorder experience more anxiety than children with PDD-NOS and autistic disorder. In line with this, we expected that ASD children with a higher (V)IQ experience more anxiety than children with a lower (V)IQ.

It is believed that this study provides new knowledge on the field of anxiety in children with ASD, because both parent- and child-rated anxiety levels were measured and these levels were compared. In the majority of the previous studies on anxiety in children with ASD [e.g., Ref. (3, 17, 30)], only parent-rated anxiety levels were measured. Since children with ASD have shown to be able to accurately report their anxious cognitions (31), self-reports of anxiety can add important valuable new knowledge about the way in which children with ASD assess their own internalizing symptoms. Moreover, there are hardly any studies that examined anxiety levels in children with ASD in Dutch children with ASD [e.g., Ref. (32)], and most studies included large community samples of children with ASD with all levels of impairment [e.g., Ref. (30)], while in this study, a specific clinical sample children with ASD was included who had a high level of daily impairment and who received intensive treatment at a mental health institute.

## Materials and Methods

### Procedures

In this study, screening data on anxiety were obtained that was later used for the inclusion of participants in a randomized controlled trial (RCT) testing the effect of an anxiety intervention for children with an ASD [see study protocol in Ref. (33)]. This RCT (including the screening procedure) was carried out with approval of the medical ethics committee “Commissie Mensgebonden Onderzoek Arnhem-Nijmegen” in the Netherlands (NL50023.091.14) and in accordance with the ethical standards of the Declaration of Helsinki as revised in 2000. Passive consent was received to use the screening data of children and parents in this study. However, when they eventually participated in the RCT and they received an intervention (experimental or control condition), active and written informed consent was received.

Specialized services in which children with ASD were recruited were two mental health institutes and one secondary special education school. First, parents received a letter with information about the study. If parents and children were interested in participation after reading the letter, they both filled in screening questionnaires on anxiety [Spence Children’s Anxiety Scale for Children (SCAS-C) (34) and Spence Children’s Anxiety Scale for Parents (SCAS-P) (35)]. All children and parents (with passive consent) who filled in screening questionnaires were part of this study, regardless of their anxiety levels and eventual participation in the RCT.

### Participants

In total, 172 children with an ASD (autistic disorder, Asperger disorder, and PDD-NOS) and their parents participated in this study. Of the participating children, 134 were males (77.9%) and 38 were females (22.1%). Their age was between 8 and 15 years (*M* = 11.25, SD = 2.02). Furthermore, 105 children were in primary school (61.0%) and 67 were in secondary school (39.0%). Of the children in primary school, 39 followed special education (37.1%). Of the children in secondary school, 38 followed special education (56.7%). Moreover, 29 children in secondary school followed vocational training (43.3%), 2 followed vocational training/high school training (3.0%), 8 followed high school training (11.9%), 15 followed high school training/pre-university training (22.4%), 6 followed pre-university training (9.0%), and 5 (7.5%) followed other secondary education. Most of the children were of Dutch origin (>90%). Inclusion criterion was sufficient knowledge of the Dutch language. Exclusion criteria were absence of parental permission and presence of prominent suicidal ideation or other severe psychiatric problems that needed immediate treatment (e.g., severe trauma).

### Instruments

#### Anxiety Symptoms

A Dutch translation of the SCAS-C (34) was used to measure anxiety symptoms of the participating children. The SCAS consists of 44 items on a 4-point scale, ranging from “never” to “always.” Scores on items range from 0 to 3, with higher scores indicating more anxiety symptoms. Moreover, the scale consists of six subscales that are in line with the different anxiety disorders that are described in the Diagnostic and Statistical Manual of Mental Disorders, fourth edition—Text Revision [DSM-IV-TR (36)]. However, the obsessive–compulsive subscale was not included in this study, because in children with ASD, this behavior is often not related to anxiety, but serves as a form of self-soothing behavior or as a regulation of excitement or arousal (37). Moreover, in the DSM-V (38), the obsessive–compulsive disorder was not defined as an anxiety disorder anymore (39). Furthermore, the fear of physical injury subscale was defined as “specific phobia” in this study. This is because the items of the SCAS-C/P that belong to this subscale do not only refer to fear of physical injury but also refer to other specific fears like fear of heights and fear of darkness. The SCAS-C contains six positive filler items, which are not used in the calculation of the total score or subscale scores. The SCAS has a high validity and reliability (32, 40). Moreover, anxiety symptoms that are measured with both the SCAS-C can be reliably clustered into the anxiety disorder categories as described in the DSM-IV (41). Cronbach’s alpha was 0.94.

Moreover, a Dutch translation of the SCAS-P (35) was used to measure anxiety symptoms of the participating children according to the parents. The SCAS-P consists of 38 items on a 4-point scale ranging from 0 (never) to 3 (always). The items of the SCAS-P were formulated as closely as possible to the corresponding item of the child version of the SCAS. The SCAS-P consists of the same six subscales as the child version. Also for the SCAS-P, the obsessive compulsive subscale was not included, and the fear of physical injury subscale was defined as “specific phobia” in this study. The SCAS-P has a good reliability and validity (42). Moreover, anxiety symptoms that are measured with both the SCAS-P can be reliably clustered into the anxiety disorder categories as described in the DSM-IV (42). Cronbach’s alpha was 0.90.

#### ASD and Comorbid Diagnoses

All the participating children were diagnosed with an ASD at a mental health institute in the Netherlands. Of these children, 119 were diagnosed with the ASD subtype PDD-NOS (69.2%), 29 were diagnosed with the ASD subtype Asperger disorder (16.9%), and 22 were diagnosed with the ASD subtype autistic disorder (12.8%). ASD diagnoses were based on the psychological and/or psychiatric assessment of the DSM-IV criteria for autistic disorder, Asperger’s disorder, or PDD-NOS. This assessment was adapted to the diagnostic “needs” of the individual child and for example consisted of a developmental anamnesis with parents, contact with the child’s teacher, and/or standardized observation of the child with the Autism Diagnostic Observation Scale (43). Comorbid diagnoses were attention deficit hyperactivity disorder (45.3%), (persistent) depressive disorder (7.0%), oppositional defiant disorder (3.5%), obsessive–compulsive disorder (1.7%), reactive attachment disorder (1.7%), and posttraumatic stress disorder (1.2%). Moreover, 9.3% of the participating children had some kind of learning disability (e.g., dyslexia).

#### IQ

The level of cognitive functioning (IQ) was measured by an intelligence test. Of the participating children, 141 children underwent an intelligence test in their clinical trajectory at the mental health institute where they were treated. The total IQ (TIQ) is an indicator of the overall level of cognitive functioning and consists of a VIQ and a PIQ. Only VIQ and PIQ were included as variables in this study. This because in this study, the mean absolute difference between VIQ and PIQ was 12.85 (SD = 9.04). A discrepancy of this magnitude is often considered to be statistically significant (44), with the consequence that TIQ was not a reliable indicator of the level of cognitive functioning of the participating children. Therefore, VIQ and PIQ could be better used as separate indicators of the level of verbal and non-verbal cognitive functioning. If children had a history of multiple IQ assessments, the most recent IQ measurement was included. In this study, the mean VIQ was M = 104.87, and the mean PIQ was M = 97.85.

In total, 134 children were assessed by the third edition of the Wechsler Intelligence Scale for Children (44), which has shown to be a reliable and valid intelligence test for children of 6–16 years old. Because two children were under the age of 6 years when they had their most recent IQ assessment, they underwent an alternative intelligence test. One child was assessed by the Revisie Amsterdamse Kinderintelligentie Test (45), and the other child was assessed by the third edition of the Wechsler Preschool and Primary Scale of Intelligence (46). Finally, five children were assessed by the non-verbal intelligence test Snijders-Oomen Niet-verbale Intelligentietest 2,5-7 (47).

### Analysis

First, the prevalence of anxiety symptoms in the participating children with ASD was assessed. Descriptive statistics (means and SDs) were retrieved from child- and parent-rated SCAS total anxiety and SCAS subscales of generalized anxiety, specific phobia, social phobia, separation anxiety, and panic disorder/agoraphobia. To assess the prevalence of elevated anxiety symptoms, the cutoffs for subclinical anxiety scores were used. By using the Dutch norm data of the SCAS-C (41) and the Dutch-Australian norm data of the SCAS-P (42), a subclinical score on total anxiety and all subscales was defined by a score of >M ± 1 SD on the SCAS-C/P. The norms for the SCAS-P were based on the means and SDs for the “normal control children” (separately for boys and girls) in the age of 6–11 years (for the children of 8–11 years) and 12–18 years (for the children of 12–15 years) that were reported in the study by Nauta et al. (42). The norms for the SCAS-C were based on the means and SDs for boys and girls in the age of 7–12 years (for the children of 8–12 years) and 13–19 years (for the children of 13–15 years) that were reported in the study by Muris et al. (41).

Second, two series of path analyses were performed using Mplus version 7.2 (48) to examine the relationship between potential risk factors and anxiety symptoms of the participating children as reported by the children (series 1) and as reported by their parents (series 2). For both series, path analyses were applied with risk factors (gender, age, VIQ, performance IQ, and ASD subtypes) as predictors and anxiety symptoms as dependent variables. By using this type of analysis, predictors were free to correlate. The advantage of path analysis above regression analysis was that all available information of the data was used. To estimate the parameters of the path model, the Bayes estimator was applied based on the Markov Chain Monte Carlo estimation using diffuse (non-informative) prior probability distributions as default. The Bayesian estimation was used, because the more traditional estimation method (Maximum Likelihood) failed due to model identification issues (49). The fit of the model was evaluated by posterior predictive checking expressed in posterior predictive *p* values (PPP). PPP values of 0.50 implied good model fit, and small PPP values (e.g., <0.05) implied poor model fit (49, 50). Standardized posterior regression coefficients were reported with Bayesian *p* values if *p* < 0.05.

The composition of ASD subtypes consisted of three categories: PDD-NOS, Asperger disorder, and autistic disorder. Effects of the three groups on anxiety symptoms and disorders were examined using unweighted effects coding (51). Two codes represented the three groups PDD-NOS, Asperger disorder, and autistic disorder; one coded 1, 0, and −1 and the second 0, 1, and −1, respectively. Regression weights represented the deviation of the outcome variable for each separate group from the grand mean. Only the effects of PDD-NOS and Asperger disorder were visible in the output. To determine the effect of autistic disorder on anxiety symptoms and disorders, a second path analysis was conducted with a different coding system (−1, 1, 0 and −1, 0, 1) (51).

### Effects of Missing Data

Because 31 children did not undergo an intelligence test (18.0% of 172), logistic regression analyses were conducted in SPSS 21 (52) to examine the attrition effects for sex and age. For the analyses on the effect of risk factors on anxiety symptoms (SCAS-C/P), attrition on VIQ and PIQ was the dependent binary variable. No differences in attrition were found for both sex (OR, 0.78; 95% CI, 0.29–2.06, *p* = 0.61) and age (OR, 1.10; 95% CI, 0.91–1.33; *p* = 0.33).

## Results

### Prevalence of Anxiety Symptoms

Table 1 shows the means and SDs of the total and subscale SCAS scores of the parents (SCAS-P) and children (SCAS-C). Table 2 shows the counts and percentages of children with at least subclinical anxiety symptoms according to parent and child assessment. In total, 66.3% of the participating children with ASD had child-rated subclinical or clinical anxiety symptoms on the total scale and/or on at least one subscale and 81.4% of the participating children with ASD had parent-rated subclinical or clinical anxiety symptoms on the total scale and/or on at least one subscale. It showed that for all anxiety disorders, more than 30% of the participating children with ASD experienced at least subclinical anxiety symptoms according to assessments of both parents and children. The parent assessment showed that for total anxiety and panic disorder/agoraphobia, more than 50% of the participating children with ASD experienced at least subclinical anxiety symptoms.

**Table 1:** Means and SDs of the Total and Subscale Scores of Children (SCAS-C) and Parents (SCAS-P).

	Children (*n* = 168), *M* (SD)	Parents (*n* = 172), *M* (SD)
Total anxiety	29.03 (18.47)	29.07 (14.61)
Separation anxiety	4.37 (3.90)	5.18 (3.62)
Social phobia	5.76 (3.92)	6.79 (3.74)
Panic disorder/agoraphobia	4.37 (4.55)	4.05 (3.42)
Specific phobia	4.20 (2.88)	4.63 (2.94)
Generalized anxiety	5.39 (3.65)	4.65 (2.61)

*n, number; M, means*.

**Table 2:** Counts, Percentages, and χ^2^(1) Values of Children with at Least Subclinical Anxiety Symptoms According to Parent and Child Assessment.

	Children (*n* = 168), *n* (%)	Parents (*n* = 168), *n* (%)	χ^2^ (1) value
Total anxiety	67 (39.9)	104 (61.9)	22.74***
Separation anxiety	60 (35.7)	80 (47.6)	7.52**
Social phobia	79 (47.0)	78 (46.4)	0.00
Panic disorder/agoraphobia	55 (32.7)	94 (56.0)	22.22***
Specific phobia	62 (36.9)	71 (42.3)	1.42
Generalized anxiety	54 (32.1)	82 (48.8)	13.50***

*n, number*.

**p ≤ 0.01.

***p ≤ 0.001.

To assess whether the difference in number of children with at least subclinical anxiety between the parent and child ratings was significant, the McNemar test for related proportions [χ*^2^*(1)] was conducted (see Table 2). The number of children with parent-rated subclinical total anxiety (*p* < 0.001), separation anxiety (*p* = 0.006), panic disorder/agoraphobia (*p* < 0.001), and generalized anxiety (*p* < 0.001) was significantly higher than the number of children with child-rated subclinical anxiety on these anxiety scales. Parent and child ratings did not significantly differ in the number of children with subclinical social phobia and specific phobia (*p* > 0.05).

### Risk Factors for Anxiety Symptoms of Children with ASD

For child-rated anxiety symptoms and parent-rated child anxiety symptoms, path analyses were conducted using the Bayes estimator. Each of the 18 path models show a fit with PPP values varying between 0.433 and 0.438, which implied a good fit (49).

### Age and Gender

The path analyses results for child-rated anxiety symptoms are shown in Table 3. Girls with ASD had significantly more total anxiety symptoms than boys with ASD (β = 0.23, *p* < 0.01). Moreover, girls with ASD had significantly more separation anxiety symptoms (β = 0.13, *p* < 0.05), social phobia symptoms (β = 0.23, *p* < 0.01), panic disorder/agoraphobia symptoms (β = 0.30, *p* < 0.001), and generalized anxiety symptoms (β = 0.19, *p* < 0.01) than boys with ASD. Gender was not a significant predictor of specific phobia symptoms.

**Table 3:** Path Analyses with Age, Gender, IQ (VIQ, PIQ), and ASD subtype (autistic disorder, Asperger disorder, and PDD-NOS) as Predictors and Child-rated Symptoms of Total Anxiety, Separation Anxiety, Social Phobia, Panic Disorder/Agoraphobia, specific phobia, and Generalized Anxiety as Dependent Variables.

	Total anxiety beta	Separation anxiety beta	Social phobia beta	Specific phobia beta	Panic/Agora phobia beta	Generalized anxiety beta
Gender	0.23**	0.13*	0.23**	0.10	0.30***	0.19**
Age	−0.14*	−0.32***	0.06	−0.08	−0.10	−0.05
VIQ	−0.07	−0.14	0.02	−0.22*	0.01	0.07
PIQ	0.07	0.08	0.01	0.20*	−0.00	−0.02
PDD-NOS	−0.01	−0.02	0.06	−0.10	0.05	0.04
AS	0.07	0.04	0.03	0.03	0.10	0.07
AD	−0.08	−0.03	−0.10	0.06	−0.19	−0.13

*Betas are standardized regression weights*.

*AD, autistic disorder; AS, Asperger disorder; PIQ, performance (non-verbal) IQ; VIQ, verbal IQ*.

*p ≤ 0.05.

**p ≤ 0.01.

***p ≤ 0.001.

Concerning the predictor age, younger children with ASD had significantly more child-rated total anxiety symptoms than older children with ASD (β = −0.14, *p* < 0.05). Also, younger children with ASD had significantly more separation anxiety symptoms (β = −0.32, *p* < 0.001) than older children with ASD. Age was not a significant predictor of social phobia symptoms, specific phobia symptoms, panic disorder/agoraphobia symptoms, and generalized anxiety symptoms.

In Table 4, the path analyses results for parent-rated child anxiety symptoms are shown. Girls with ASD had significantly more total anxiety symptoms than boys with ASD (β = 0.17, *p* = 0.01). Moreover, girls with ASD had significantly more social phobia symptoms (β = 0.21, *p* < 0.01), panic disorder/agoraphobia symptoms (β = 0.22, *p* < 0.01), and generalized anxiety symptoms (β = 0.22, *p* < 0.01) than boys with ASD. Gender was not a significant predictor of separation anxiety symptoms and specific phobia symptoms.

**Table 4:** Path Analyses with Age, Gender, IQ (VIQ, PIQ), and ASD subtype (autistic disorder, Asperger disorder, and PDD-NOS) as Predictors and Parent-rated Symptoms of Total Anxiety, Separation Anxiety, Social Phobia, Panic Disorder/Agoraphobia, Specific Phobia, and Generalized Anxiety as Dependent Variables.

	Total anxiety beta	Separation anxiety beta	Social phobia beta	Specific phobia beta	Panic/agora phobia beta	Generalized anxiety beta
Gender	0.17**	0.06	0.21**	−0.01	0.22**	0.22**
Age	−0.09	−0.27***	0.10	−0.15*	−0.03	−0.06
VIQ	−0.02	0.01	0.05	−0.07	−0.08	−0.04
PIQ	0.14	0.12	0.07	0.14	0.15	0.14
PDD-NOS	−0.09	−0.04	0.04	−0.11	−0.16*	−0.06
AS	−0.15	−0.05	−0.20**	−0.10	−0.08	−0.09
AD	0.28**	0.10	0.22*	0.25*	0.27**	0.17

*Betas are standardized regression weights*.

*AD, autistic disorder; AS, Asperger disorder; PIQ, performance (non-verbal) IQ; VIQ, verbal IQ*.

*p ≤ 0.05.

**p ≤ 0.01.

***p ≤ 0.001.

Concerning the predictor age, younger children with ASD had significantly more parent-rated separation anxiety symptoms (β = −0.27, *p* < 0.001) and specific phobia symptoms (β = −0.15, *p* < 0.05) than older children with ASD. Age was not a significant predictor of total anxiety, social phobia symptoms, panic disorder/agoraphobia symptoms, and generalized anxiety symptoms.

### IQ

Children with a lower VIQ had significantly more child-rated specific phobia symptoms than children with a higher VIQ (β = −0.22, *p* < 0.05), and children with a higher PIQ had significantly more specific phobia symptoms than children with a lower PIQ (β = 0.20, *p* < 0.05; see Table 3). VIQ and PIQ were not found to be significant predictors of all other child-rated subscale anxiety symptoms. According to parents, VIQ and PIQ were not significant predictors of all parent-rated total and subscale anxiety symptoms (see Table 4).

### ASD Subtypes

For child-rated anxiety, none of the three ASD subtypes were significant predictors of all child-rated total and subscale anxiety symptoms (see Table 3). For parent-rated anxiety, children with PDD-NOS had significantly less panic disorder/agoraphobia symptoms (β = −0.16, *p* < 0.05) compared to the overall mean of this variable (see Table 4). Moreover, children with Asperger disorder had significantly less social phobia symptoms (β = −0.20, *p* = 0.01) compared to the overall mean of this variable. Children with autistic disorder had significantly more total anxiety symptoms (β = 0.28, *p* < 0.01), more social phobia symptoms (β = 0.22, *p* < 0.05), more specific phobia symptoms (β = 0.25, *p* < 0.05), and more panic disorder/agoraphobia symptoms (β = 0.27, *p* = 0.01) compared to the overall means of these variables.

## Discussion

The first aim of this study was to provide an overview of the prevalence of anxiety symptoms in a clinical Dutch sample of children with ASD. The findings showed that the prevalence of both parent-rated and child-rated subclinical and clinical anxiety symptoms was high: more than 60% of the participating children with ASD had at least subclinical anxiety symptoms according to children and more than 80% of the children had at least subclinical anxiety symptoms according to parents. Parents reported higher total anxiety, separation anxiety, panic disorder/agoraphobia, and generalized anxiety than children. The results of this study provide new knowledge on the field of anxiety in children with ASD, because child-rated anxiety levels were measured, and these levels were compared with parent-rated anxiety levels, while in the majority of the previous studies on anxiety in children with ASD [e.g., Ref. (3, 17, 30)], only parent-rated anxiety levels were measured. Moreover, there are hardly any studies that examined anxiety levels in Dutch children with ASD [e.g., Ref. (32)], and most studies included large community samples of children with ASD with all levels of impairment [e.g., Ref. (30)], while in this study, a specific clinical sample children with ASD was included who had a high level of daily impairment and who received intensive treatment at a mental health institute.

The child-rated prevalence of anxiety (66.3%) that was found in this study was only slightly higher than the prevalence rates in the study of Strang et al. (3) and Vasa et al. (17). They found that 56 (3), 52 (school children) (17), and 54% (adolescents) (17) of the children with ASD had at least subclinical anxiety symptoms. However, other studies found that only 22 (8) and 46% (30) of the children with ASD had at least subclinical anxiety symptoms. Moreover, the parent-rated prevalence (81.4%) of anxiety that was found in this study was higher than the prevalence rates that were found in all the above described studies (3, 8, 17, 30).

There are several possible explanations for the relatively high prevalence rates in this study. The prevalence rates of anxiety may differ as a result of the differences in levels of cognitive functioning between the participants included in the studies. The participating children in the study by Gotham et al. (30) had, for example, a mean VIQ of 80 and a mean non-VIQ of 86. The children who participated in this study had a relatively high mean VIQ and non-VIQ of, respectively, 105 and 98, and higher IQ has shown to be related to higher levels of anxiety [e.g., Ref. (1, 20)].

Moreover, it is possible that the differences in prevalence rates are due to the use of different cutoffs for anxiety symptoms. Strang et al. (3), for example, reported prevalence rates of subclinical anxiety on the basis of a borderline score on a general anxiety scale of a questionnaire, while in this study, subclinical anxiety was defined as a subclinical score on the total scale and/or on at least one subscale of the SCAS-C/P. When the prevalence rates of subclinical anxiety were only based on the total SCAS scale, 43.3% of the children had child-rated subclinical anxiety symptoms and 63.4% of the children had parent-rated subclinical anxiety symptoms in this study. These percentages are more comparable to those that were found in other studies (3, 17, 30).

It is also possible that the difference in cultural background of the participants in this study, and previous studies could be an explanation for the differences in outcomes. Several studies showed that SCAS-based anxiety scores differ among different cultures and countries (53, 54). Since previous studies predominantly or entirely included American participants and mostly used American norm data [e.g., Ref. (30)] and this study included only Dutch participants and used Dutch norm data (SCAS-C) and Dutch–Australian norm data (SCAS-P), it is possible that the difference in origin and cultural background partly explained the difference in reported prevalence of anxiety in children with ASD.

Finally, anxiety levels may differ as a result of the differences in sample specificity. The study with lower prevalence rates of anxiety (30) included a large community sample of children with ASD, with anxiety levels that may not have reached clinical significance in all children. This study included a clinical Dutch sample with ASD children, who received treatment at a mental health institute because of the high daily impairment that in most cases was (partly) caused by the presence of high anxiety levels.

The higher parent ratings compared to the child ratings of anxiety could be explained by the family problems that were highly prevalent in this study. These problems could lead to less agreement between parents and children in their report of anxiety. Parents with conflicts, stress, or psychiatric symptoms may project their own problems on their children, leading to a parental overreport of anxiety symptoms (55). These discrepancies suggest that it is important to use both child and parent report in studies on children with ASD.

The second aim was to investigate the risk factors for anxiety in this sample of children with ASD. More specifically, age, gender, ASD subtype, and IQ were investigated as risk factors for anxiety in children with ASD. Parent-rated total anxiety symptoms were more prevalent among younger than among older children with ASD. This finding contradicts previous studies that have shown that anxiety increases with age in the ASD population [e.g., Ref. (18, 19)]. The findings in this study are more in line with research in the normal population showing that most anxiety disorders decrease with age, because childhood anxiety may lead to other psychopathology in adolescence, for example, depression (56). This similarity to the findings in the normal population may be due to the relatively high cognitive levels of the participating children compared to other studies in the ASD population (4), which may be related to a course of psychopathology levels over time that is also present in the normal population (18). When examining the findings on age differences in the prevalence of separation anxiety and specific phobia symptoms in children with ASD, it can be concluded that these findings are also in line with previous research in the normal population. This research indicated early childhood as a risk period for separation anxiety and specific phobia and showed a decrease in prevalence from early childhood to mid adolescence (57, 58).

Concerning the differential prevalence rates of anxiety for boys and girls with ASD, girls with ASD had more anxiety than boys with ASD. This finding was not in line with previous research showing that there are no gender differences in anxiety between boys and girls with ASD (17, 21, 22). However, the finding that girls with ASD are more anxious than boys with ASD is consistent with research on gender differences in anxiety in the normal population (59). Again, this may be explained by the relatively high cognitive levels of the ASD sample in this study compared to other studies (4), leading to the same neuropsychological basis of gender differences in anxiety as in the normal population (23). Moreover, the higher prevalence of anxiety in girls in this study could be explained by the higher acceptability of female anxiety by parents (60), which may have led to underreport of clinical anxiety in boys by parents. Moreover, having ASD is related to higher stress levels (61), which in turn leads to a more profound expression of personality traits in terms of coping strategies. Anxiety-related personality traits (e.g., neuroticism) are more prevalent among girls than among boys (60), which suggest that girls with ASD show more anxious behavior and coping strategies than boys with ASD.

The finding that ASD children with a higher PIQ and lower VIQ had more child-rated specific phobia symptoms is inconsistent with research showing that deficits in both receptive and expressive communication skills are related to less anxiety symptoms in children with ASD (28), but is consistent with the finding that children with PDD-NOS had higher anxiety levels as communication deficits increased (29). There is some evidence that specific fears related to particular people, animals, or situations could be reduced by verbalization of feelings (62), which may prevent the development of this kind of anxiety into a full specific phobia. ASD children with a higher PIQ and lower VIQ may have difficulty with verbalizing their feelings, explaining why they experience more symptoms of specific phobia. Other anxiety disorders may be more complex in nature, which may have led to a smaller, non-significant impact of these verbalization skills on the presence of these anxiety disorders.

On one hand, the finding that parent-rated anxiety is more prevalent in children with autistic disorder than in children with Asperger disorder and PDD-NOS contradicts some previous studies [e.g., Ref. (2, 9, 25)], but, on the other hand, this finding is more or less in line with other studies [e.g., Ref. (4, 19)]. A possible explanation for the inconsistent findings on ASD subtype as risk factor for anxiety is the limited validity in how ASD subtypes are assigned in the DSM-IV, with similar core symptom presentations across subtypes (63). This limited specificity of subtypes may have led to the variation in anxiety levels in children with different ASD subtypes between the existing studies. The low validity of the ASD subtypes in the DSM-IV was the rationale for moving to a categorical system in the new DSM-V with a single diagnostic dimension: severity of ASD symptoms (38).

There are some limitations in this study. The intelligence levels of the participating children were not always known, leading to a considerably smaller number of children that could be included in this part of the analyses. Despite the estimations of the missing values that were made in the analyses, these missing values could have lowered the reliability of the statistical results. Moreover, the questionnaires (SCAS-C/P) in this study were not specially designed for children with ASD (2). Also, it is possible that not only pure anxiety symptoms were measured with the SCAS, but also impairments that are related to the core ASD symptoms (e.g., social withdrawal as a consequence of high arousal). As a consequence, the participating children with ASD may not have accurately reported their anxiety symptoms. In their review on the use of anxiety assessment tools in children with ASD, Grondhuis and Aman (64) reported that the lack of psychometric characteristics for the ASD population of the SCAS may lead to an unreliable assessment of anxiety. However, they also state that the SCAS might be useful for ASD children who are higher functioning and who have moderate language and cognitive abilities (64), which are characteristics that are applicable to the participating children in the present study. Moreover, it is possible that not only the presence of an ASD was related to the high level of anxiety symptoms in this study but also other comorbid disorders such as attention deficit hyperactivity disorder, which has also shown to be highly related to anxiety in children [e.g., Ref. (65)]. Finally, the lack of a comparison group and the use of norms could possibly have biased the outcomes in this study.

One clear message from this study is the high extent to which anxiety problems co-occur with ASD in a clinical sample of children. When children with ASD are referred to a mental health institute, they often have subclinical or clinical levels of anxiety. However, what we do not yet know is the developmental sequelae of these disorders and the impact each has on the other over time. Future research using longitudinal designs to investigate the mechanisms responsible for the development of anxiety in children with ASD seems warranted. Moreover, it is important to investigate the reliability and validity of the new ASD classifications in the DSM-V and the predictive validity of anxiety questionnaires in the population of children with ASD, because this may lead to less sample and methodological heterogeneity. These suggested future studies could eventually lead to a more extensive, more valid, and more reliable explanatory framework for the development of anxiety in children with ASD.

This study has some implications for clinical practice. The findings suggest that children with ASD have a high risk to have co-occurring anxiety symptoms. Especially girls and younger children with ASD may be at risk to develop anxiety. Therefore, early prevention of anxiety in children with ASD who are most at risk is important. On basis of the results of this study, it seems especially important to offer an anxiety prevention program to young girls (e.g., in the age of 4–8 years) with ASD. Moreover, this study showed that a considerable number of children with ASD already met the criteria of one or more anxiety disorders. Currently, anxiety is often interpreted as a part of the ASD in clinical practice, which may lead to an underreporting and thus lack of treatment for anxiety as a separate condition (“diagnostic overshadowing” (66)). When anxiety in children with ASD is treated, this may also lead to a decrease in impairment caused by the core ASD symptoms (2). Thus, attention has to be paid to the assessment of the presence of anxiety disorders when children with ASD are referred to or receive mental health care. When children with ASD meet the criteria for one or more anxiety disorders, it is important that they receive treatment for their anxiety symptoms that is adapted to their capacities and difficulties.

## Ethics Statement

The present study was carried out in accordance with the ethical standards of the medical ethics committee CMO Arnhem-Nijmegen in the Netherlands (NL50023.091.14) and with the Declaration of Helsinki as revised in 2000. Specialized services in which children with ASD were recruited were two mental health institutes and one secondary special education school. All parents agreed with the anonymous usage of their data for scientific purposes when they entered the mental health institute or special education school.

## Author Contributions

LW is responsible for the data collection, the data analysis, and reporting the study results. DC, AV, and IG are supervisors, grant applicators, and principal investigators. All authors have contributed to the writing of this manuscript. Moreover, all authors have read and approved the final manuscript.

## Conflict of Interest Statement

The authors declare that the research was conducted in the absence of any commercial or financial relationships that could be construed as a potential conflict of interest.

## References

[1] Salazar F Baird G Chandler S Tseng E O’sullivan T Howlin P Co-occurring psychiatric disorders in preschool and elementary school-aged children with autism spectrum disorder. J Autism Dev Disord (2015) 45(8):2283–94. 10.1007/s10803-015-2361-5 25737019

[2] White SW Oswald D Ollendick t Scahill L Anxiety in children andadolescents with autism spectrum disorders. Clin Psychol Rev (2009) 29:216–29. 10.1016/j.cpr.2009.01.003 19223098 PMC2692135

[3] Strang JF Kenworthy L Daniolos P Case L Wills MC Martin A Depression and anxiety symptoms in children and adolescents with autism spectrum disorders without intellectual disability. Res Autism Spectr Disord (2012) 6:406–12. 10.1016/j.rasd.2011.06.015 22615713 PMC3355529

[4] Van Steensel FJA Bögels SM Perrin S Anxiety disorders in children andadolescents with autistic spectrum disorders: a meta-analysis. Clin Child Fam Psychol Rev (2011) 14:302–17. 10.1007/s10567-011-0097-0 21735077 PMC3162631

[5] Kerns CM Kendall PC The presentation and classification of anxiety in autism spectrum disorder. Clin Psychol SciPract (2012) 19:323–47. 10.1111/cpsp.12009

[6] Bradley EA Summers JA Wood HL Bryson SE. Comparing rates of psychiatric and behavior disorders in adolescents and young adults with severe intellectual disability with and without autism. J Autism Dev Disord (2004) 34(2):151–61. 10.1023/b:jadd.0000022606.97580.19 15162934

[7] Bellini S Social skill deficits and anxiety in high-functioning adolescents with autism spectrum disorders. Focus Autism Other Dev Disabl (2004) 19(2):78–86. 10.1177/10883576040190020201

[8] Lecavalier L. Behavioral and emotional problems in young people with pervasive developmental disorders: relative prevalence, effects of subject characteristics, and empirical classification. J Autism Dev Disord (2006) 36(8):1101–14. 10.1007/s10803-006-0147-5 16897387

[9] Gadow KD Devincent CJ Pomeroy J Azizian A. Comparison of DSM-IV symptoms in elementary school-age children with PDD versus clinic and community samples. Autism (2005) 9(4):392–415. 10.1177/1362361305056079 16155056

[10] Meyer JA Mundy PC Van Hecke AV Durocher JS. Social attribution processes and comorbid psychiatric symptoms in children with Asperger syndrome. Autism (2006) 10(4):383–402. 10.1177/1362361306064435 16908481 PMC2654174

[11] Bauminger N Kasari C Brief report: theory of mind in high-functioning children with autism. J Autism Dev Disord (1999) 29(1):81–6. 10.1023/a:1025974701090 10097997

[12] De Bruin EI Ferdinand RF Meester S de Nijs PFA Verheij F. High rates of psychiatric co-morbidity in PDD-NOS. J Autism Dev Disord (2007) 37(5):877–86. 10.1007/s10803-006-0215-x 17031447

[13] Drahota A Wood JJ Sze KM van Dyke M Effects of cognitive behavioral therapy on daily living skills in children with high functioning autism and concurrentanxiety disorders. J Autism Dev Disord (2011) 41(3):257–65. 10.1007/s10803-010-1037-4 20508979 PMC3040302

[14] Hallett V Lecavalier L Sukhodolsky DG Cipriano N Aman M McCracken JT Exploring the manifestations ofanxiety in children with autism spectrum disorders. J Autism Dev Disord (2013) 43(10):2341–52. 10.1007/s10803-013-1775-1 23400347 PMC4038127

[15] Kim JA Szatmari P Bryson SE Streiner DL Wilson FJ The prevalence of anxiety and mood problems among children with autism and Asperger syndrome. Autism (2000) 4(2):117–32. 10.1177/1362361300004002002

[16] Cervantes P Matson JL Tureck K Adams HL The relationship of comorbid anxiety symptom severity and challenging behaviors in infants and toddlers with autism spectrum disorder. Res Autism Spectr Disord (2013) 7:1528–34. 10.1016/j.rasd.2013.09.005

[17] Vasa RA Kalb L Mazurek M Kanne S Freedman B Keefer A Age-related differences in the prevalence and correlates of anxiety in youth with autism spectrum disorders. Res Autism Spectrum Disord (2013) 7:1358–69. 10.1016/j.rasd.2013.07.005

[18] Davis TE III Hess JA Moree BN Fodstad JC Dempsey T Jenkins WS Anxiety symptoms across the lifespan in people diagnosedwith autistic disorder. Res Autism Spectr Disord (2011) 5:112–8. 10.1016/j.rasd.2010.02.006

[19] Dickerson Mayes SD Calhoun SL Murray MJ Zahid J Variables associated with anxiety and depression in children with autism. J Dev Phys Disabil (2011) 23:325–37. 10.1007/s10882-011-9231-7

[20] Dubin AH Lieberman-Betz R Michele Lease A. Investigation of individual factors associated with anxiety in youth with autism spectrum disorders. J Autism Dev Disord (2015) 45(9):2947–60. 10.1007/s10803-015-2458-x 25917383

[21] Sukhodolsky DG Scahill L Gadow KD Arnold LE Aman MG McDougle CJ Parent-rated anxiety symptoms in children with pervasive developmental disorders: frequency and association with core autism symptoms and cognitive functioning. J Abnorm Child Psychol (2008) 36:117–28. 10.1007/s10802-007-9165-9 17674186

[22] Worley JA Matson JL Sipes M Koziowski AM Prevalence of autism spectrum disorders in toddlers receiving early intervention services. Res Autism Spectrum Disord (2010) 5:920–5. 10.1016/j.rasd.2010.10.007

[23] Brereton AV Tonge BJ Einfeld SL. Psychopathology in children and adolescents with autism compared to young people with intellectual disability. J Autism Dev Disord (2006) 36(7):863–70. 10.1007/s10803-006-0125-y 16897401

[24] MacNeil BM Lopes VA Minnes PM Anxiety in children and adolescents with autism spectrum disorders. Res Autism Spectr Disord (2009) 3(1):1–21. 10.1016/j.rasd.2008.06.001

[25] Weisbrot D Gadow K DeVincent C Pomeroy J. The presentation of anxiety inchildren with pervasive developmental disorders. J Child Adolesc Psychopharmacol (2005) 15:477–96. 10.1089/cap.2005.15.477 16092912

[26] Blackshaw AJ Kinderman P Hare DJ Atton CH. Theory of mind, causal attribution and paranoia in Asperger syndrome. Autism (2001) 5:147–63. 10.1177/1362361301005002005 11706863

[27] Chapman NH Estes A Munson J Bernier R Webb SJ Rothstein JH Genome-scan for IQ discrepancy in autism: evidence for loci on chromosomes 10 and 16. Hum Genet (2011) 129(1):59–70. 10.1007/s00439-010-0899-z 20963441 PMC3082447

[28] Davis TE III Moree BN Dempsey T Hess JA Jenkins WS Fodstad JC The effect of communication deficits on anxiety symptoms in infants and toddlers with autism spectrum disorders. Behav Ther (2012) 43(1):142–52. 10.1016/j.beth.2011.05.003 22304886

[29] Davis TE III Moree BN Dempsey T Reuther ET Fodstad JC Hess JA The relationship between autism spectrum disorders and anxiety: the moderating effect of communication. Res Autism Spectr Disord (2011) 5(1):324–9. 10.1016/j.rasd.2010.04.015

[30] Gotham K Bishop SL Hus V Huerta M Lund S Buja A Exploring the relationship between anxiety and insistence on sameness in autism spectrum disorders. Autism Res (2013) 6(1):33–41. 10.1002/aur.1263 23258569 PMC4373663

[31] Ozsivadjian A Hibberd C Hollocks MJ Brief report: the use of self-report measures in young people with autism spectrum disorder to access symptoms of anxiety, depression and negative thoughts. J Autism Dev Disord (2014) 44(4):969–74. 10.1007/s10803-013-1937-1 24014195

[32] Muris P Steerneman P Merckelbach H Holdrinet I Meesters C. Comorbid anxiety symptoms in children with pervasive developmental disorders. J Anxiety Disord (1998) 12(4):387–93. 10.1016/S0887-6185(98)00022-X 9699121

[33] Wijnhoven LAMW Creemers DHM Engels RCME Granic I The effect of the video game ‘mindlight’ on anxiety symptoms of children with an autism spectrum disorder: study protocol for a randomized controlled trial. BMC Psychiatry (2015) 15:138 10.1186/s12888-015-0522-x 26129831 PMC4488062

[34] Scholing A Nauta MH Spence SH Spence Children’s Anxiety Scale (DutchTranslation of Child Version). Amsterdam, NL: University of Amsterdam (1999).

[35] Scholing A Nauta MH Spence SH Spence Children’s Anxiety Scale (DutchTranslation of Parent Version). Amsterdam, NL: University of Amsterdam (1999).

[36] American Psychiatric Association. Diagnostic and Statistical Manual of Mental Disorders. (Vol. 4). Washington, DC: APA (2000).

[37] Scahill L Challa SA Repetitive behavior in children with autism spectrum disorder: similarities and differences with obsessive-compulsive disorder. In: Mazzone L Vitiello B, editors. Psychiatric Symptoms and Comorbidities in Autism Spectrum Disorder. Cham: Springer International Publishing (2016). p. 39–50.

[38] American Psychiatric Association. Diagnostic and Statistical Manual of Mental Disorders. (Vol. 5). Washington, DC: APA (2013).

[39] Van Ameringen M Patterson B Simpson W. Dsm-5 obsessive-compulsive and related disorders: clinical implications of new criteria. Depress Anxiety (2014) 31(6):487–93. 10.1002/da.22259 24616177

[40] Spence SH Barrett PM Turner CM Psychometric properties of the Spence Children’s Anxiety Scale with young adolescents. J Anxiety Disord (2003) 17:605–25. 10.1016/s0887-6185(02)00236-0 14624814

[41] Muris P Schmidt H Merckelbach H Correlations among two self-report questionnaires for measuring DSM-defined anxiety disorder symptoms in children:the screen for child anxiety related emotional disorders and the Spence Children’s Anxiety Scale. Pers Individ Dif (2000) 28:333–46. 10.1016/s0191-8869(99)00102-6

[42] Nauta MH Scholing A Rapee R Abbott M Spence SH Waters AA Parent-report measure of children’s anxiety: psychometric properties and comparison with child-report in a clinic and normal sample. Behav Res Ther (2004) 42:813–39. 10.1016/S0005-7967(03)00200-6 15149901

[43] Bildt A Greaves-Lord K De Jonge M ADOS-2 Nederlandse Bewerking. [ADOS-2 Dutch Adaptation]. Amsterdam: Hogreve uitgevers (2013).

[44] Wechsler D WISC-III: Wechsler Intelligence Scale for Children: 3rd Edition. SanAntonio, TX: Psychological Corporation (1991).

[45] Bleichrodt N Drenth PJD Zaal JN Resing WCM Revisie Amsterdamse Kinderintelligentie Test (RAKIT). Lisse: Swets & Zeitlinger (1987).

[46] Wechsler D WPPSI-III Administration and Scoring Manual. San Antonio, TX: Psychological Corporation (2002).

[47] Tellegen P Winkel M Wijnberg-Williams B Laros J Snijders-Oomen Niet Verbale Intelligentietest, SON-R 21/2-7. Lisse: Swets & Zeitlinger (1998).

[48] Muthén LK Muthén BO Mplus User’s Guide. Seventh Edition. Los Angeles, CA: Muthén & Muthén (1998).

[49] Zyphur MJ Oswald FL Bayesian estimation and inference: a user’s guide. J Manag (2015) 41(2):390–420. 10.1177/0149206313501200

[50] Muthén B Asparouhov T. Bayesian structural equation modeling: a more flexible representation of substantive theory. Psychol Methods (2012) 17(3):313–35. 10.1037/a0026802 22962886

[51] Cohen J Cohen P West SG Aiken LS Applied Multiple Regression/Correlation Analysis for the Behavioral Sciences (3rd Ed.). Mahwah, New Jersey: Lawrence ErlbaumAssociates (2003).

[52] IBM Corp. IBM SPSS Statistics for Windows, Version 21.0. Armonk, NY: IBM Corp (2012).

[53] Li JB Delvecchio E Di Riso D Nie YG Lis A. The parent-version of the Spence Children’s Anxiety Scale (SCAS-P) in Chinese and Italian community samples: validation and cross-cultural comparison. Child Psychiatry Hum Dev (2016) 47(3):369–83. 10.1007/s10578-015-0572-9 26289082

[54] Essau CA Sasagawa S Anastassiou-Hadjicharalambous X Guzman BO Ollendick TH. Psychometric properties of the Spence Child Anxiety Scale with adolescents from five European countries. J Anxiety Disord (2011) 25(1):19–27. 10.1016/j.janxdis.2010.07.001 20685072

[55] Kroes G Veerman JW De Bruyn EEJ Bias in parental reports? Maternal psychopathology and the reporting of problem behavior in clinic-referred children. Eur J Psychol Assess (2003) 19(3):195–203. 10.1027//1015-5759.19.3.195

[56] Seligman LD Ollendick TH. Comorbidity of anxiety and depression in children and adolescents: an integrative review. Clin Child Fam Psychol Rev (1998) 1(2):125–44. 10.1023/a:1021887712873 11324302

[57] Breton J-J Bergeron L Valla J-P Berthiaume C Gaudet N Lambert J ‘Quebec child mental health survey: prevalence of DSM-III-R mental health disorders’. J Child Psychol Psychiatry (1999) 40(3):375–84. 10.1111/1469-7610.00455 10190339

[58] Kashani JH Orvaschel H. A community study of anxiety in children and adolescents. Am J Psychiatry (1990) 147(3):313–8. 10.1176/ajp.147.3.313 2309948

[59] Lewinsohn PM Gotlib IH Lewinsohn M Seeley JR Allen NB. Gender differences in anxiety disorders and anxiety symptoms in adolescents. J Abnorm Psychol (1998) 107(1):109–17. 10.1037/0021-843X.107.1.109 9505043

[60] McLean CP Anderson ER. Brave men and timid women? A review of the gender differences in fear and anxiety. Clin Psychol Rev (2009) 29(6):496–505. 10.1016/j.cpr.2009.05.003 19541399

[61] Gillott A Standen P. Levels of anxiety and sources of stress in adults with autism. J Intellect Disabil (2007) 11(4):359–70. 10.1177/1744629507083585 18029412

[62] Tabibnia G Lieberman MD Craske MG. The lasting effect of words on feelings: words may facilitate exposure effects to threatening images. Emotion (2008) 8(3):307–17. 10.1037/1528-3542.8.3.307 18540747 PMC4727455

[63] Grzadzinski R Huerta M Lord C. DSM-5 and autism spectrum disorders (ASDs): an opportunity for identifying ASD subtypes. Mol Autism (2013) 4(1):12. 10.1186/2040-2392-4-12 23675638 PMC3671160

[64] Grondhuis SN Aman MG Assessment of anxiety in children and adolescents with autism spectrum disorders. Res Autism Spectr Disord (2012) 6(4):1345–65. 10.1016/j.rasd.2012.04.006

[65] Jarrett MA Wolff JC Davis TE Cowart MJ Ollendick TH. Characteristics of children with ADHD and comorbid anxiety. J Atten Disord (2016) 20:636–44. 10.1177/1087054712452914 22863769

[66] Simonoff E Pickles A Charman T Chandler S Loucas T Baird G. Psychiatric disorders in children with autism spectrum disorders: prevalence, comorbidity, and associated factors in a population-derived sample. J Am Acad Child Adolesc Psychiatry (2008) 47(8):921. 10.1097/CHI.0b013e318179964f 18645422

